# Transatlantischer Handelskonflikt und die deutsche Wirtschaft: Auf die Dauer kommt es an

**DOI:** 10.1007/s10273-020-2694-3

**Published:** 2020-07-18

**Authors:** Sebastian Dullien, Sabine Stephan, Thomas Theobald

**Affiliations:** 1 in der Hans-Böckler-Stiftung, Inst. f. Makroökonomie u. Konjunkturf. (IMK), Georg-Glock-Str. 18, 40474 Düsseldorf, Deutschland; 2 in der Hans-Böckler-Stiftung: Ökonometrie, Inst. f. Makroökonomie u. Konjunkturf. (IMK), Georg-Glock-Str. 18, 40474 Düsseldorf, Deutschland; 3 in der Hans-Böckler-Stiftung: Finanzmärkte und Konjunktur, Inst. f. Makroökonomie u. Konjunkturf. (IMK), Georg-Glock-Str. 18, 40474 Düsseldorf, Deutschland

**Keywords:** E17, F13, F17

## Abstract

Auch in der Corona-Krise schwelen internationale Handelskonflikte weiter. So verschärfte die US-Regierung Mitte Mai 2020, mitten in der Corona-Krise, noch einmal die Exportbeschränkungen für Zulieferer des chinesischen Telekommunikationsausrüsters Huawei. Angesichts des dramatischen Nachfrageeinbruchs bei den Automobilherstellern kann deshalb auch nicht ausgeschlossen werden, dass Donald Trump seine protektionistische Idee, die Einfuhr von Autos und Autoteilen aus der EU mit Strafzöllen zu belegen, wiederbeleben und damit auch den transatlantischen Handelskonflikt neu anfachen könnte. Simulationen zeigen, dass ein solcher Konflikt insbesondere bei einer Ausweitung der Zölle auf weitere Sektoren und einer längeren Dauer der deutschen Wirtschaft spürbaren Schaden zufügen könnte.

## Abstract (en)

International trade conflicts continue to simmer in times of the coronavirus crisis. Most recently, in mid-May, the US government tightened export restrictions for suppliers to the Chinese technology company Huawei. Against the background of the dramatic slump in consumer demand for automobiles, US President Donald Trump might revive his protectionist idea of tariffs on cars and car parts from the EU, escalating the transatlantic trade conflict. Economic policy simulations show that such an escalation has the potential to dampen the economic recovery in Germany after the pandemic shock. Fiscal rules limit the ability to counterbalance the macroeconomic impact of a trade war, putting Germany potentially at a relative disadvantage in this conflict.

Bereits im vergangenen Jahr und vor Ausbruch der Corona-Krise wurden die internationalen Handelskonflikte immer wieder als ein wesentlicher Faktor für die Abschwächung des deutschen Wirtschaftswachstums und als Risikofaktor für die kommenden Monate genannt (Dullien et al., 2019a; Projektgruppe Gemeinschaftsdiagnose, 2019). Neben den indirekten Effekten (etwa dadurch, dass Unternehmen in China infolge des Handelskriegs mit den USA weniger Maschinen in Deutschland bestellen) galt die Aufmerksamkeit insbesondere der Drohung der USA, den Zollsatz für die Einfuhr von Kraftfahrzeugen und -teilen aus der EU auf 25 % anzuheben.

**Prof. Dr. Sebastian Dullien** lehrt Volkswirtschaftslehre an der Hochschule für Technik und Wirtschaft Berlin und ist Wissenschaftlicher Direktor des Instituts für Makroökonomie und Konjunkturforschung in der Hans-Böckler-Stiftung (IMK) in Düsseldorf.

**Dr. Sabine Stephan** leitet das Referat Ökonometrie im IMK.

**Dr. Thomas Theobald** ist wissenschaftlicher Referent für Finanzmärkte und Konjunktur im IMK.

Zwar entspannte sich Mitte November 2019 die Lage etwas, als US-Präsident Trump die Frist verstreichen ließ, in der er nach US-Recht aus Gründen der „nationalen Sicherheit“ die Autozölle hätte verhängen können. Aber bereits Anfang Dezember drohte die US-Regierung im Streit um die französische Digitalsteuer wieder mit Strafzöllen und brachte wenig später Autozölle im Konflikt um den europäischen Umgang mit dem Iran ins Gespräch. Anfang Juni legte Trump dann noch einmal nach und drohte der EU mit Autozöllen, sollte diese nicht die Zölle auf US-amerikanische Hummer senken. Die Gefahr eskalierender Handelskonflikte ist also mitnichten gebannt.

Zölle auf Autos sind aus deutscher Sicht besonders sensibel. Zum einen, weil der Automobilbau zu den wichtigsten deutschen Industriebranchen gehört. Zum anderen, weil mit dem Maschinenbau und der Chemieindustrie zwei weitere zentrale Wirtschaftszweige als Zulieferer stark von der Automobilindustrie abhängen. Da die deutsche Wirtschaft 2020 wegen der Corona-Krise massiv schrumpfen dürfte, Haushalte unter Einkommensverlusten leiden und Unternehmen Liquidität und Eigenkapital verloren haben, würde ein sich zuspitzender Handelskonflikt der USA mit der EU ein Hemmnis für eine zügige Erholung der Wirtschaft nach der Corona-Krise darstellen.

Der vorliegende Beitrag verwendet das makroökonometrische Globalmodell des National Institute (NiGEM),[1] um die möglichen Folgen einer Anhebung der US-Einfuhrzölle auf Fahrzeuge und -teile aus der EU sowie einer Verschärfung des transatlantischen Handelskonflikts auf die gesamtwirtschaftliche Entwicklung in Deutschland zu un-

1 Wir verwenden das Zollmodell v19.2t2.

tersuchen.[2] Angelehnt an die Dramaturgie des Handelskriegs zwischen den USA und China werden verschiedene Szenarien simuliert, die aufeinander „gestapelt“ eine zunehmende Verschärfung des transatlantischen Handelskonflikts abbilden. Auf diese Weise können die gesamtwirtschaftlichen Effekte unterschiedlicher Eskalationsstufen – vom Zollscharmützel bis zum ausgewachsenen Handelskrieg – abgeschätzt werden.

Des Weiteren hat die US-Regierung vor der Corona-Krise einiges unternommen, um die negativen Auswirkungen aktueller Handelskonflikte auf die US-Wirtschaft abzufedern. So wurden Branchen, die von chinesischen Vergeltungsmaßnahmen besonders stark betroffen waren, mit umfangreichen staatlichen Beihilfen kompensiert (Parker und Dorning, 2019). Deswegen wird in einem zweiten Schritt untersucht, wie die gesamtwirtschaftlichen Auswirkungen eines Handelskonflikts dadurch beeinflusst werden können, dass ihn die Kontrahenten mit expansiven fiskalpolitischen Maßnahmen flankieren.

## Transmissionskanäle einer Zollerhöhung

Bei der Konzeption der Simulation ist zunächst zu überlegen, welche Wirkungszusammenhänge bei einem Handelskonflikt eine Rolle spielen und wie diese in NiGEM abgebildet werden können. Im Zentrum der Betrachtung steht zunächst der Importzoll, dessen Wirkungsweise von der Marktstruktur und der Größe des Landes abhängt, das den Zoll einführt.

Ein kleines Land ist auf dem Weltmarkt Preisnehmer, d. h., dass inländische Konsumenten jede beliebige Menge des Importguts zum Weltmarktpreis erwerben können, ohne dass sich dadurch der Preis ändert. Wenn inländische Verbraucher heimische und ausländische Erzeugnisse als vollkommene Substitute betrachten, ist die Importmenge die Differenz zwischen der Menge, die im Inland von heimischen Produzenten zum Weltmarktpreis angeboten wird, und der Gesamtmenge, die von den Verbrauchern zu eben diesem Preis nachgefragt wird.

Ein Einfuhrzoll erhöht unter diesen Bedingungen den inländischen Verkaufspreis des importierten Produkts genau um den Zoll. Das bedeutet, dass der Zoll vollständig an die Verbraucher weitergegeben wird, die ihre Nachfrage entsprechend reduzieren. Da auch der Preis für das heimische Substitut steigt, dehnen inländische Firmen ihre Produktion aus, und die Verbraucher weichen zunehmend auf das inländische Substitut aus. Während inländische Produzenten nach der Einführung des Importzolls eine größere Menge zu einem höheren Preis verkaufen können, müssen ausländische Exporteure, die weiterhin zum unveränderten Weltmarktpreis anbieten, einen Absatzrückgang hinnehmen. Dieser Rückgang ist jedoch nicht groß genug, um sich auf den Weltmarktpreis des betreffenden Guts auszuwirken.

Wenn ein großes Land[3] einen Einfuhrzoll erhebt, ist der Mechanismus etwas anders: Auch hier gilt, dass eine Erhöhung des Importzolls den Preis im Inland erhöht, sodass die nachgefragte Menge sinkt. In diesem Fall führt jedoch der Nachfragerückgang des Inlands auf dem Weltmarkt dazu, dass der Weltmarktpreis des betreffenden Guts sinkt. Das bedeutet, dass die ausländischen Exporteure einen geringeren Preis erzielen und der Inlandspreis um weniger als den Zoll steigt. Die zollbedingte Preisänderung wird somit nicht mehr vollständig von den inländischen Verbrauchern getragen, sondern in gewissem Umfang von den ausländischen Exporteuren, die neben dem Mengenrückgang auch Preissenkungen hinnehmen müssen.

Aufgrund der ökonomischen Zusammenhänge setzen viele Simulationsstudien, auch mit NiGEM, Zölle in der Regel über Änderungen der Import- und Exportpreise um (Deutsche Bundesbank, 2018), was im Modellzusammenhang dann dazu führt, dass die Verbraucherpreise in dem Land, das den Zoll erhebt, steigen. Gleichwohl bildet diese Modellierungsstrategie nur einen der möglichen Transmissionskanäle ab. Je nach Marktstruktur kann es andere Wirkungszusammenhänge geben.

Die Marktform der vollkommenen Konkurrenz ist zur Beschreibung der Märkte vieler handelbarer Güter (und auch des Automobilmarkts) eher ungeeignet; besser eignet sich hier die Marktform der monopolistischen Konkurrenz (Krugman, 1979). Dieses Konzept geht davon aus, dass es viele Anbieter gibt, deren Produkte nicht identisch sind und daher von den Verbrauchern nur als unvollkommene Substitute betrachtet werden. Dies hat zwei wichtige Konsequenzen: Erstens sind die Unternehmen auf solch einem Markt zu einem gewissen Grad Preissetzer und können deshalb strategische Preissetzung (Pricing to market) betreiben. Zweitens erzielen diese Unternehmen beständig Gewinne, weil sie ihre Produkte mit einem – zum Teil beträchtlichen – Preisaufschlag oberhalb ihrer Grenzkosten verkaufen.

Wird nun ein Zoll auf Produkte verhängt, die in solch einem Markt verkauft werden, dann beeinflusst das die exportierenden Unternehmen wie folgt: Erstens können sie in Abhängigkeit von der Preiselastizität der Nachfrage und

2 Dullien et al. (2019b) präsentieren vergleichbare Ergebnisse für die Eurozone und die USA.

3 „Groß“ in dem Sinne, dass seine globale Nachfrage nach dem betreffenden Gut groß genug ist, um den Weltmarktpreis zu beeinflussen.

dem Wettbewerbsdruck auf dem jeweiligen Absatzmarkt entscheiden, in welchem Umfang sie den Zoll als Preisaufschlag an ihre Konsumenten weitergeben oder ob sie den Zoll in der eigenen Gewinnmarge absorbieren, zumal wenn sie davon ausgehen, dass der Zoll nur vorübergehend verhängt wird. Zweitens führt selbst eine partielle Weitergabe zu zusätzlichen Verlusten, da sie einen sinkenden Marktanteil zur Folge hat; jede Einheit des Produkts wird nämlich zu einem Absatzpreis oberhalb der Grenzkosten verkauft. Im Gegenzug bedeutet die Erhebung eines Zolls auf diesem Markt, dass sich die Gewinnsituation für inländische Unternehmen verbessert: Wenn etwa Autohersteller aus der EU für ihre Verkäufe auf dem US-Markt Zölle zahlen müssen und ihre Preise – wenn auch nicht um den vollen Betrag des Zolls – erhöhen, wird der Wettbewerbsdruck auf die US-Hersteller abnehmen. Diese können nun mehr Einheiten verkaufen oder auch ihre Preise erhöhen, was sich beides in steigenden Gewinnen niederschlägt (Krugman, 1979; Helpman und Krugman, 1989).

Ein weiterer Wirkungskanal besteht aufgrund der internationalen Produktionsverflechtungen. Die von den USA angedrohten Autozölle betreffen nicht nur fertig montierte Kraftfahrzeuge, sondern auch Kraftfahrzeugteile. Deutsche Automobilhersteller betreiben große Werke in den USA und verbauen dort auch Teile, die aus der EU und insbesondere aus Deutschland zugeliefert wurden. Ein Einfuhrzoll auf Kfz-Teile erhöht die Produktionskosten deutscher Autobauer in den USA und verschlechtert ihre preisliche Wettbewerbsfähigkeit gegenüber Konkurrenten und zwar sowohl auf dem US-Automarkt als auch auf Drittmärkten. Denn anders als andere ausländische Autohersteller fertigen deutsche Autokonzerne in den USA nicht nur für den US-Markt, sondern in großem Umfang auch für den Export. BMW und Daimler sind die beiden größten Autoexporteure der USA.

Zusammenfassend wird deutlich, dass US-Autozölle die Gewinne deutscher Autohersteller unmittelbar schmälern würden. Dadurch könnten auch die Finanzierungskosten dieser Unternehmen auf den Kapitalmärkten steigen und bei einer Strategie konstanter Dividendenzahlungen die für Innovationen und Investitionen zur Verfügung stehenden Mittel reduziert werden. Eine solche Folge des Handelskonflikts lässt sich in NiGEM durch eine Erhöhung der von den Kreditgebern geforderten Risikoprämie modellieren.

Schließlich können Handelskonflikte zu einer zunehmenden ökonomischen Unsicherheit führen, da die Dauer der Auseinandersetzung und ihr Ausgang ungewiss sind und sich Unternehmen deshalb mit Investitionen zurückhalten. Auch dies lässt sich im Modell mit einer Erhöhung der Risikoprämie abbilden (OECD, 2019).

Jenseits der beschriebenen Wirkungskanäle sind weitere denkbar, auf deren Modellierung jedoch bewusst verzichtet wurde, weil es empirisch nicht klar erscheint, dass sie aufgrund des Handelskonflikts zum Tragen kommen. In diesem Zusammenhang sind Wechselkursanpassungen zu nennen. Erlaubt man – entgegen der hier gewählten Vorgehensweise – eine modellendogene Anpassung des Außenwerts des Euro, dann würden steigende US-Zölle auf Verbraucherpreise und Zinsen durchwirken, was tendenziell zu einer Aufwertung des US-Dollar führen würde. Wird daraufhin Kapital aus anderen Wirtschaftsräumen, insbesondere aus Schwellenländern, abgezogen, dann drohen die mit dem Handelskonflikt einhergehenden globalen Wachstumsverluste noch gravierender auszufallen als in den dargestellten Simulationen (Erken et al., 2018). Ähnliches gilt, wenn man annimmt, dass die Zolleinführung für Exportnationen Produktivitätsverluste auslöst – sei es in Form eines Verlust an Kapitalproduktivität (Krugman, 2018) oder an Arbeitsproduktivität (Erken et al., 2018). Dementsprechend können die im Folgenden präsentierten Ergebnisse als Untergrenze möglicher makroökonomischer Auswirkungen aufgefasst werden.

## Makroökonometrische Simulationen

Da der Verlauf eines Handelskonflikts nicht vorhersehbar ist, ist es sinnvoll, verschiedene Szenarien zu untersuchen. Zu diesem Zweck werden drei Simulationsansätze mit unterschiedlichen Annahmen hinsichtlich der Dauer des Handelskonflikts und der Reaktion der Fiskalpolitik getroffen und mit jeweils vier Szenarien kombiniert, die eine Eskalation des Handelskonflikts abbilden (vgl. Tabelle 1).

Bei einem *kurzen Handelskonflikt* (1) nehmen wir an, dass die USA Einfuhrzölle auf Produkte aus der EU nur für eineinhalb Jahre erhöht. Diese Annahme basiert auf der Möglichkeit, dass der jetzige US-Präsident im November 2020 abgewählt wird und eine neue Regierung im Anschluss die Rücknahme der protektionistischen Handelspolitik einleitet. Bei einem kurzen Handelskonflikt wird zudem angenommen, dass die Exporteure die Kosten der Zölle nicht an die Verbraucher weitergeben, sondern in ihrer Gewinnmarge absorbieren.

Bei einem *langen Handelskonflikt* (2) wird eine Fortsetzung der Zwistigkeiten über die US-Präsidentschaftswahl hinaus unterstellt. In diesem Fall dürfte die Zollpolitik die Handelsbeziehungen zwischen den USA und der EU für die nächsten fünf Jahre bestimmen. Den Exporteuren dürfte es zunehmend schwerer fallen, die Kosten der Zölle allein zu tragen. Es wird daher angenommen, dass die Zölle voll auf die Verbraucherpreise durchwirken (Amiti et al., 2019). Eine Gegenüberstellung der beiden Simulationsergebnisse erlaubt sodann Rückschlüsse auf den Fall, dass die zugrunde liegenden Annahmen nur teilweise erfüllt sind (mittlere Geltungsdauer der Zölle, teilweise Überwälzung der Kosten).

Beim *langen Handelskonflikt mit fiskalpolitischer Stabilisierung* (3) wird angenommen, dass sowohl die USA als auch – in geringerem Maße – die EU-Staaten Kredite aufnehmen, um die heimische Wirtschaft durch expansive Fiskalpolitik zu stützen. Diese Annahme beruht auf Beobachtungen im Handelsstreit zwischen den USA und China. So hat die US-Regierung die von diesem Handelskonflikt besonders betroffenen Farmer bereits mit beträchtlichen staatlichen Beihilfen unterstützt und noch weitere in Aussicht gestellt (Parker und Dorning, 2019).

In den drei Simulationsansätzen werden jeweils vier Szenarien (A bis D) gerechnet, die sich am bisherigen Verlauf und den weltwirtschaftlichen Konsequenzen des Handelskonflikts zwischen den USA und China orientieren: (A) Die USA verhängen Zölle auf die Einfuhr von Kraftfahrzeugen und -teilen aus der EU. (B) Die USA weiten die Einfuhrzölle auf weitere Produkte aus der EU aus. (C) Die EU reagiert auf die Eskalation der USA mit entsprechenden Gegenmaßnahmen. (D) Der Handelskonflikt zwischen den großen Wirtschaftsräumen belastet die Weltwirtschaft dadurch, dass die Unsicherheit der Unternehmen hinsichtlich zukünftiger Absatzmöglichkeiten zunimmt.

Die Eskalation des Handelskonflikts wird dadurch abgebildet, dass die Szenarien aufeinander „gestapelt" werden, d. h., dass mit jeder weiteren Simulation ein Schock auf die bereits simulierten Schocks aufgesattelt wird. Die Größe der Schocks in den Szenarien A bis D lässt sich wie folgt motivieren: Autos und Kfz-Teile machen im EU-Durchschnitt 10 % der Warenexporte in die USA aus; vor allem für Deutschland liegt dieser Anteil mit 20 % aber beträchtlich höher. Im Autozoll-Szenario (A) übersetzt sich daher ein Zollsatz in Höhe von 25 % auf Kraftfahrzeuge und -teile aus der EU in einen Anstieg der US-Importpreise für Produkte aus Deutschland um 5 % und in einen Anstieg der US-Importpreise für Güter aus der übrigen EU um 2,5 %. Im erweiterten Zollszenario (B) werden die Strafzölle nach einem halben Jahr auf weitere Produkte ausgeweitet, sodass der Anstieg der US-Importpreise für Produkte aus allen EU-Ländern dann einheitlich 10 % beträgt. Reagiert die EU auf die Ausweitung der US-Importzölle mit Gegenmaßnahmen (C), steigen die EU-Importpreise für Produkte aus den USA ebenfalls um 10 %. Um die Auswirkungen der Handelsstreitigkeiten auf die ökonomische Unsicherheit (D) zu modellieren, wird für den Fall des kurzen Handelskonflikts eine Erhöhung der Risikoprämie für kreditfinanzierte Investitionen in den direkt vom Handelsstreit betroffenen Regionen um 40 Basispunkte angenommen. Der unterstellte Anstieg ist vergleichbar mit den Annahmen in anderen aktuellen Studien (z. B. OECD, 2019).[4] Im Fall eines lang andauernden Handelskonflikts wird ein fast doppelt so starker Anstieg um 75 Basispunkte und eine Ausbreitung der Unsicherheit auf die übrigen Wirtschaftsräume der Welt angenommen. Diese Annahme lässt sich mit den schon beobachteten wirtschaftlichen Folgen des Handelskonflikts der USA mit China motivieren.

Tabelle 1
**Aufbau der kontrafaktischen Simulationen**

| Simulation | Simulationsansatz | Schockdauer |
|---|---|---|
| 1 | Kurzer Handelskonflikt: Exporteure überwälzen die Zölle nicht auf Konsumenten | 7/2020 bis 12/2021 |
| 2 | Langer Handelskonflikt: Exporteure überwälzen die Zölle auf Konsumenten | 7/2020 bis 12/2025 |
| 3 | Langer Handelskonflikt mit fiskalpolitischer Stabilisierung | 7/2020 bis 12/2025 |
| **Szenario** | **Annahmen zur Entwicklung des Handelskonflikts zwischen den USA und der EU[1]** | **gültig ab** |
| A | US-Autozölle: 25 % auf Auto-Importe aus der EU im Wert von 45 Mrd. Euro | 7/2020 |
| B | US-Zollausweitung: 25 % auf Importe aus der EU im Gesamtwert von 160 Mrd. Euro | 1/2021 |
| C | EU-Zölle auf Importe aus den USA, Zollsatz und Umfang entsprechen den US-Maßnahmen | 1/2021 |
| D | Handelskonflikt verursacht Unsicherheit: Risikoprämie steigt um 40 bzw. 75 Basispunkte | 7/2020 |

[1] Die Szenarien werden „gestapelt", um in dem jeweiligen Simulationsansatz die Eskalation des Handelskonflikts abzubilden.

Quelle: Zusammenstellung des IMK.

## Simulationsergebnisse

### Kurzer Handelskonflikt

In den reinen Zollszenarien (A bis C) beschränken sich die Effekte weitgehend auf den Außenhandel (vgl. Abbildung 1). Da die deutschen Exporteure die US-Einfuhrzölle weitgehend in ihren Gewinnmargen absorbieren, steigen ihre Exportpreise nur leicht, sodass die Exportvolumen nur moderat sinken. Dass auch die Importe zurückgehen, liegt maßgeblich an dem hohen Importgehalt deutscher Exporte. Die deutschen Verbraucherpreise bleiben in den reinen Zollszenarien annähernd konstant, sodass bei unveränderter Kaufkraft auch der private Verbrauch konstant bleibt. In dem Fall, dass die EU ihrerseits Einfuhrzölle auf US-Produkte erhebt, steigt der private Verbrauch im Vergleich zum Basisszenario ohne Handelskonflikt sogar leicht, weil der deutsche Staat diese Zolleinnahmen – per angenom-

4 Dieser Anstieg betrifft ausschließlich den Fremdfinanzierungsanteil der Investitionen. Da nicht gleichzeitig angenommen wird, dass die Eigenkapitalgeber höhere Risikoprämien verlangen, relativiert dies die unterstellte Höhe der Unsicherheitsschocks.

mener Fiskalregel – an die Haushalte zurückgibt und damit deren Kaufkraft stärkt. Die reinen Zollszenarien haben so gut wie keine Auswirkungen auf die Investitionsnachfrage hierzulande. Insgesamt fällt die gesamtwirtschaftliche Entwicklung in Deutschland im Vergleich zum Basisszenario ohne Handelskonflikt nur leicht schwächer aus.

Wenn in der Simulation zusätzlich zu den Zollmaßnahmen eine erhöhte ökonomische Unsicherheit berücksichtigt wird (Szenario D), ergibt sich ein deutlich anderes Bild. Die ökonomische Unsicherheit führt zu einem Einbruch der Investitionsnachfrage in Deutschland, mit der Konsequenz, dass die Nachfrage nach importierten Investitionsgütern ebenfalls deutlich sinkt. Da die Unsicherheit auch die globale Nachfrage schwächt, gehen die Exporte zurück. Dies führt wegen des hohen Importgehalts deutscher Exporte dazu, dass die Importnachfrage noch weiter sinkt.

Unter dem Strich geht das BIP in Deutschland verglichen mit dem Basisszenario ohne Handelskonflikt spürbar zurück, was zu einem leichten Anstieg der Arbeitslosenquote führt. Allerdings unterschätzt die hier verwendete Version des NiGEM-Modells den Einfluss der aggregierten Nachfrage auf die Arbeitslosigkeit, da es einen gegenläufigen Effekt gibt. Bei einer neoklassischen Modellierung des Arbeitsmarkts stabilisieren schwächere Lohnanstiege die Beschäftigung. Eine aktualisierte Schätzung der NiGEM-Beschäftigungsgleichung weist demgegenüber darauf hin, dass der Einfluss der Löhne auf die Beschäftigung weniger stark negativ ausfällt (Behrend, 2019). Die hier aufgeführten Ergebnisse können deshalb als Untergrenze der makroökonomischen Auswirkungen aufgefasst werden.

## Langer Handelskonflikt

In den reinen Zollszenarien (A bis B) beschränken sich die negativen Effekte zunächst auf den Außenhandel (vgl. Abbildung 2). Da die deutschen Exporteure in einem lang andauernden Handelskonflikt mit den USA die Zollerhöhungen an die Endverbraucher überwälzen, steigen ihre Exportpreise mit der Konsequenz, dass das Exportvolumen kräftig sinkt. Da die deutschen Exporte einen hohen Importgehalt haben, kommt es gleichzeitig zu einem nennenswerten Rückgang des Importvolumens, sodass der Außenhandel per Saldo das BIP nur wenig belastet. Die gesamtwirtschaftlichen Einbußen werden etwas größer, wenn in Szenario C die US-Exporteure die EU-Strafzölle an die Verbraucher in der EU weitergeben, weil dann die Importe und infolge der leicht anziehenden Inflation auch Exporte und Investitionen noch etwas stärker zurückgehen. In der Simulation, die zusätzlich zu den Zollmaßnahmen die erhöhte ökonomische Unsicherheit berücksichtigt (Szenario D), kommt es zu einem regelrechten Einbruch der Investitionen hierzulande. Der Rückgang der globalen

Abbildung 1
**Makroökonomische Auswirkungen eines kurzen Handelskonflikts in Deutschland**

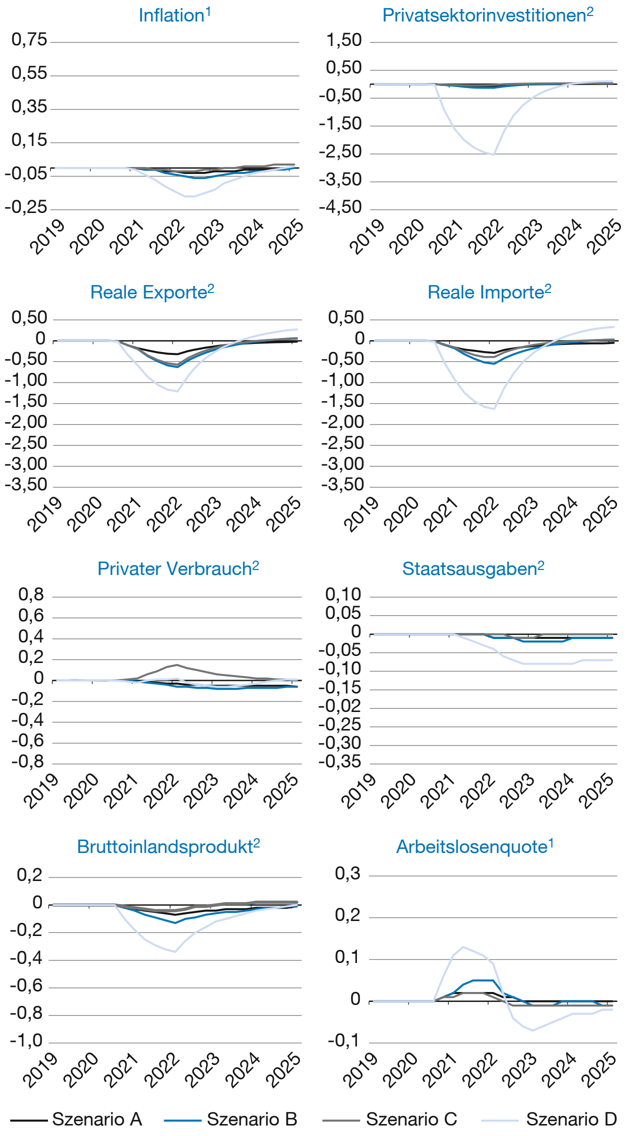

[1] Absolute Abweichung gegenüber der Basis. [2] Abweichung in % gegenüber der Basis.

Quelle: Berechnungen des IMK unter Verwendung von NiGEM.

Nachfrage führt dazu, dass die Exporte und aufgrund des hohen Importgehalts auch die Importe nochmals deutlich zurückgehen. In der ersten Hälfte des Simulationszeitraums erleidet Deutschland signifikante Wachstumsverluste, die in der zweiten Hälfte etwas korrigiert werden. Da die Inflation sinkt und der deutsche Staat die Zolleinnahmen – per angenommener Fiskalregel – an die Verbraucher weitergibt, nimmt die Kaufkraft der privaten Haushalte in der zweiten Hälfte des Simulationszeitraums etwas zu, sodass der private Verbrauch das BIP wieder stabilisiert.

Abbildung 2
**Makroökonomische Auswirkungen eines langen Handelskonflikts in Deutschland**

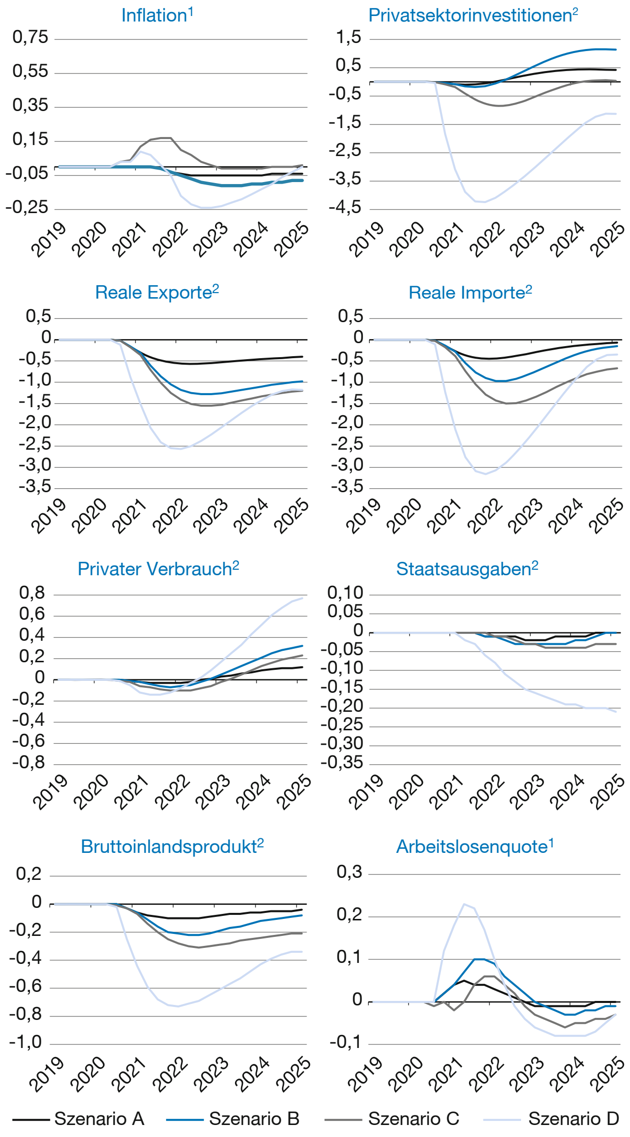

[1] Absolute Abweichung gegenüber der Basis. [2] Abweichung in % gegenüber der Basis.

Quelle: Berechnungen des IMK unter Verwendung von NiGEM.

In der Spitze geht das BIP gegenüber dem Basisszenario um 0,7 % zurück; der damit einhergehende Anstieg der Arbeitslosigkeit um etwas mehr als 0,2 Prozentpunkte dürfte den tatsächlichen Effekt aus den oben genannten Gründen deutlich unterschätzen.

## Langer Handelskonflikt mit fiskalpolitischer Stabilisierung

Die Simulationsergebnisse legen nahe, dass ein lang anhaltender Handelskonflikt nicht nur für die unmittelbar involvierten Volkswirtschaften schmerzhaft wäre, sondern auch die Konjunktur in der übrigen Welt in Mitleidenschaft ziehen würde. Wie aber passt das zu der Tatsache, dass die bestehenden Handelskonflikte die gesamtwirtschaftliche Entwicklung in den USA bis zum Ausbruch der Corona-Krise vergleichsweise wenig beeinträchtigt haben? Ein wichtiger Grund ist die expansive Fiskalpolitik in den USA seit 2018. Ein weiterer besteht darin, dass die US-Regierung einiges unternimmt, um Branchen, die besonders stark vom Handelskrieg betroffen sind, mit umfangreichen staatlichen Beihilfen zu unterstützen.[5] Damit stellt sich die Frage, inwieweit Länder einen Handelskrieg „gewinnen" können, indem sie ihn mit einer expansiven Fiskalpolitik flankieren.

Abbildung 3
**Makroökonomische Auswirkungen eines langen Handelskonflikts mit fiskalischer Stabilisierung**

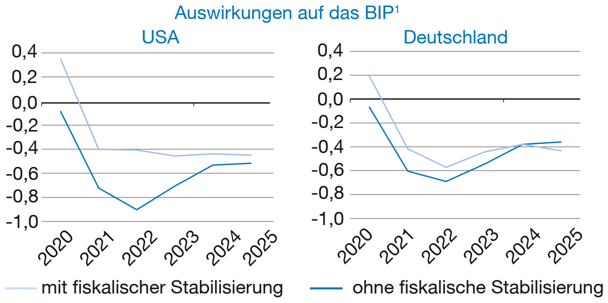

[1] Abweichung in % gegenüber der Basis.

Quelle: Berechnungen des IMK unter Verwendung von NiGEM.

Diese Frage wird im Rahmen des dritten Simulationsansatzes untersucht. Dafür wird die Simulation des lang anhaltenden Handelskonflikts um die Annahme ergänzt, dass die USA und die EU-Staaten ihre Staatsausgaben schuldenfinanziert erhöhen, um ihre Volkswirtschaften zu stabilisieren. Die Voraussetzungen für eine derartige Wirtschaftspolitik sind in den USA und in den EU-Ländern denkbar verschieden; sowohl, was Entschlossenheit zur Nutzung dieses wirtschaftspolitischen Instruments angeht, als auch, was den nationalen Handlungsspielraum hinsichtlich Haushaltsdefizit und Staatsverschuldung betrifft. Aufgrund der Reaktionen der Fiskalpolitik in den vergangenen Abschwüngen, aber auch wegen der EU-Regeln sowie nationaler Regeln zur Begrenzung der Neuverschuldung etwa im Rahmen der deutschen Schuldenbremse, kann angenommen werden, dass eine solche Stabilisierung in den USA entschlossener ausfällt als in Europa. Deshalb wird für die USA eine kreditfinanzierte Erhöhung der Staatsausgaben in Höhe von 1 %

5 2018 hatte die US-Regierung ein Nothilfe-Paket für US-Farmer in Höhe von 12 Mrd. US-$ auf den Weg gebracht. Im Mai 2019 kündigte US-Präsident Trump weitere Beihilfen im Wert von 16 Mrd. US-$ an.

des BIP[6] modelliert, während der entsprechende Impuls für die EU-Länder nur 0,5 % des BIP beträgt.

Abbildung 3 zeigt die Auswirkungen eines langen Handelskonflikts ohne und mit fiskalpolitischer Flankierung auf die gesamtwirtschaftliche Entwicklung in den USA und in Deutschland. Betrachtet wird das sehr wahrscheinliche Szenario D, in dem der Handelskonflikt alle Eskalationsstufen durchläuft und die Unsicherheit in der Wirtschaft stark erhöht. Insgesamt wird der schädliche Effekt des Handelskonflikts auf das Wirtschaftswachstum in beiden Wirtschaftsräumen abgemildert, wenngleich, wie zu erwarten, in unterschiedlichem Ausmaß. In den USA führt der entschlossenere fiskalische Impuls dazu, dass der BIP-Rückgang im Unsicherheitsszenario in der Spitze nur etwa halb so groß ausfällt wie im Fall ohne Stabilisierung. In Deutschland, das in der Simulation einen weniger expansiven fiskalischen Impuls setzt, fallen die Wachstumsverluste in der Spitze nur um etwa ein Fünftel geringer aus als im Fall ohne Stabilisierung. Zudem verschieben sich die relativen Verluste: Zwar ist auch der kräftige fiskalpolitische Impuls in den USA nicht in der Lage, die negativen Auswirkungen des Handelskriegs auszugleichen. Aber das BIP in den USA stabilisiert sich deutlich schneller auf einem erträglichen Niveau von -0,4 % gegenüber dem Basisszenario. Deutschland, dessen Wachstumsverluste in einem langen Handelskonflikt ohne fiskalpolitische Stabilisierung geringer waren als die der USA, gerät nun im Vergleich zu den USA leicht ins Hintertreffen.

## Schlussfolgerungen

Zusammenfassend lässt sich festhalten, dass ein lang anhaltender Handelskonflikt selbst bei einer fiskalpolitischen Stabilisierung spürbaren Schaden in den beteiligten Wirtschaftsräumen anrichtet. Dabei ist es vor allem die vom Handelskonflikt verursachte Unsicherheit, die über gedämpfte Investitionen das Wachstum bremst. Akzeptiert die Wirtschaftspolitik den Wachstumsdämpfer, so kann dieser groß genug ausfallen, um die Erholung der deutschen Wirtschaft in der aktuellen Krise merklich zu verzögern.

Bei einer – wie von uns simulierten – asymmetrischen fiskalpolitischen Reaktion kann es dabei den USA gelingen, hinsichtlich der relativen Wachstumsverluste als Gewinner aus dem Handelskonflikt hervorzugehen. Würde die Bundesregierung auf ein fiskalpolitisches Gegensteuern vollkommen verzichten, weil sie z. B. nach den Stützungsmaßnahmen in der Corona-Krise keinen finanzpolitischen Handlungsspielraum mehr sieht, während die Trump-Regierung die leidtragenden Sektoren des Handelskonflikts im eigenen Land mit staatlichen Beihilfen schadlos hält, dann dürfte der Schaden für Deutschland spürbar größer sein als für die USA.

6 Der fiskalpolitische Impuls wird jeweils bis zum Ende des Simulationszeitraums 2025 aufrechterhalten. Aufgrund der Größe des Impulses für die USA erfolgt die Erhöhung in zwei Stufen (Anfang 2020 und Mitte 2021). Das hat zur Folge, dass die simulierten BIP-Verläufe für die USA auf Quartalsbasis nicht kontinuierlich sind, sondern Mitte 2021 einen Sprung aufweisen. Dieser Sprung würde entfallen, wenn die Anhebung des Impulses auf 1 % des BIP kontinuierlich erfolgen würde. Eine solche Umsetzung wäre in der Praxis allerdings vollkommen unrealistisch. Um die Ausschläge in den Zeitreihen auf Quartalsbasis zu glätten, werden in Abbildung 3 Jahreswerte gezeigt.
